# A New Scheme for Infant Milk Depôts

**Published:** 1905-06

**Authors:** J. M. Fortescue-Brickdale

**Affiliations:** Physician to Out-patients, and Pathologist at the Royal Hospital for Sick Children, Bristol


### A NEW SCHEME FOR INFANT MILK DEPÔTS.

BY

J. M. FORTESCUE-BRICKDALE, M.A., M.D.,

*Physician to Out-patients, and Pathologist at the Royal Hospital for Sick Children, Bristol.*

In a former paper[1] I have given some account of the infant milk depôts in this country, and have commented to a certain extent on their methods and the results they have obtained. In the present paper I propose to extend my comments a little, and to make certain suggestions which have occurred to me in the course of an examination into the working of these institutions. In the first place I think it must be admitted that a large amount of money has been spent, and that considerable annual deficits are likely to continue in the working of the existing milk depôts, and that the results, as far as the infant mortality rate is concerned, have not been as yet very startling. It is, in fact, very generally held that there is no reliable statistical evidence forthcoming in support of milk depôts. This, apart from the necessary complexity of the problem, is no doubt partly due to the short time during which milk depôts have been working, which renders the figures liable to error from various sources, and is tantamount to admitting that milk

[1] *Bristol Med.-Chir. J.*, vol. xxii., Sept., 1904, p. 196.

depôts are still in the experimental stage. To take an instance: at Liverpool the number of children fed at the depôts from 1901 to 1903 was 6,295, while every year at least 20,000 children were born within the city.[1] Many of these children were very ill when they first took the milk, many took it for a short time, and many were irregular customers, hence it is hardly surprising that the effect on the infant mortality rate was not very apparent. It is true that the rate fell during these three years till in 1903 it stood at 159, but in 1904 it rose to 196, and no doubt both the decline and the rise are to be attributed largely to meteorological conditions. On the other hand, it can be shown that the death rate among depôt-fed infants generally is a low one. In Liverpool last year it was only 88.25, in spite of many adverse circumstances; but figures like these are also open to criticism, owing to the varying social circumstances of the infants and their parents.

The evidence available in support of the system, therefore, over and above any *primâ facie* case which can be made out for it, lies in the general opinion formed by those who have had experience of its working, and this on the whole may be said to be favourable. The difficulty of obtaining satisfactory statistical evidence lies largely in the fact that a milk depôt is after all exactly what the French call it, a *goutte de lait* in an ocean of bad milk, bad methods, and bad mothers.

It has occurred to me that, although no reliable statistics can in the nature of things be expected as to an experiment of this sort till after the lapse of some years, the experimental stage might be considerably shortened, better statistics might be attained, and several other advantages secured if the area of supply of a milk depôt were strictly limited, instead of being, as at present, spread over a city containing perhaps many hundred thousand inhabitants. It is the way in which lying-in charities are usually worked, and I cannot help feeling that the utility of these institutions might even now be seriously doubted if they had undertaken to attend all cases occurring within the city in which they were established instead of only those in their immediate vicinity.

[1] The actual number of births during 1904 was 24,277.

I propose now to outline the details of a scheme for the establishment of a milk depôt with a limited area of distribution, and to show the advantages which I think such a scheme would possess.

Such a milk depôt should be regarded, to a certain extent, as an *experimental* charity; that is to say, it should be regarded as a test institution, and every care should therefore be taken to make the conclusions to be drawn from it reliable. It should not, however, be forgotten that, even if it were found that the results obtained were no more conclusive than those at present available, the milk depôt could not fail to do a certain amount of good. As Dr. Hope has remarked in dealing with the figures obtained at Liverpool, what would the 4,453 infants fed at the Corporation depôts[1] have been fed on if they had not got this milk? It is clear that some good must be done, and the point to be decided is whether it is proportionate to the cost. Seeing, therefore, that it is in the nature of an experiment, and that its benefits will be restricted to a small area of the city in which it is established, I do not think it would be justifiable to finance such a depôt out of the rates—at all events entirely. My own view is that the capital for starting it should be raised by private subscription, and that the annual deficit should be covered by a guarantee fund. The guarantee fund should be divided into a number of equal shares, each share rendering the holder liable up to a certain amount; so that if there were fifty guarantors holding two shares each of £1 and fifty holding one share each of £1, and the deficit came to £75, the holders of two shares would each be called on for £1 and the holders of one share for 10s.

I think that the control of such a depôt should be vested in a committee having a majority of medical members, and that the general practitioners resident in the area to be supplied should be fully represented. This would ensure that efficient medical control which has been considered so necessary by many writers on the subject. The area should be carefully chosen; it should be one in which statistics of the infant mortality during

[1] This number is exclusive of infants supplied through agents.

recent years can be ascertained, in which this mortality has been consistently high, and in which the proportion of well-to-do people is small. It should not, however, be an area in which the majority of the mothers are in daily employment at factories or workshops, as such districts are unsuitable for anything but crêches, in which the infants are cared for during the day as well as fed. The statistical advantages of this plan would be that owing to the limited area a close supervision could be kept over the cases, those unsuitable socially could be more easily excluded or allowed for in the tables of results, and after a few years a more accurate comparison would be possible. There would also be certain advantages in working, which will now be enumerated.

(1) *Economy.*—There would be a certain economy in the original capital expenditure. The area being limited, and the highest number of children that would possibly have to be provided for being known, the plant and machinery would not have to be constructed to meet an enormous possible increase. At York, for instance, the plant is capable of dealing with 400 babies, and at the end of the first year's working there were about seventy on the books. At Liverpool the sterilisers can supply over 1,000 infants per diem, and during the whole year 1904 only just over 2,000 were supplied, about 500 being on the books at a time. On the other hand, at Battersea, during the summer of 1903 the resources of the depôt were severely taxed owing to the number of mothers who applied for the milk from places outside the borough. In working expenses the main item in which economy is possible seems to be wages. The experience of milk depôts is that a manageress and one assistant, with a little occasional help, are enough for about 100 infants. If an area were chosen in which the number of births amounted to perhaps 200 per annum, the wages to be paid would not be liable to increase owing to an influx of customers from outlying districts, many of whom would have very little effect on the results to be deduced from the working at the end of the year, while adding considerably to the expense. By placing the control in private hands perhaps some general economy of working would result. The fact that the sources of income were strictly limited would tend to produce care in detail, and no additional expenditure would be possible beyond the amount of the guarantee fund. In addition to this a certain income might be raised by selling the milk at a profit to well-to-do people. Residence within the area being a necessity for obtaining the milk at a cheap rate, there would be no possibility of those who could afford to pay a profitable price abusing the charity, while a committee directly interested in the depôt would be able to make it known among the wealthier classes.

(2) In a large city it is difficult to obtain sufficient notoriety for a milk depôt among the class which it is intended to benefit. Advertisement would be much easier in a small district, especially if the general practitioners and charitable institutions therein could be interested in its success.

(3) The distribution of the milk could also be made easier and more effective. One of the chief obstacles to the general use of the milk depôts by the poor is the daily journey to the depôt for the purpose of fetching the milk. If the area were limited a cart could deliver the milk at comparatively little extra cost to the management; and this would also prevent the milk being used by persons outside the prescribed radius. The system of agencies could also be avoided. This would be undoubtedly an advantage, as the supply of milk through the agency of local shopkeepers is unsatisfactory in at least two ways. Very little supervision is possible over the infants so supplied, as their addresses are generally unknown, and the milk is liable to be kept too long or even directly tampered with by the agent. There would, however, be no objection to an agency in a residential district for selling the depôt milk at a profitable price.

The actual cost of such a milk depôt would be about £200, including the necessary alterations to premises to make them suitable for the purpose.[1] It is difficult to estimate the working expenses, and consequently the deficit, but the latter would

[1] Dr. Robertson, the Medical Officer of Health for Leith, in a private letter to the writer, puts the cost of establishing a small milk depôt at £150, but this would hardly allow for any expenses in alterations to premises.

probably amount to about £100 per annum. Much would depend on the extent of the sales at a profitable price to wealthier people. The charges to the latter might be five shillings a week, as this sum, after deducting a commission to the agent, would yield a very fair profit. With regard to the charges to persons living in the district, it would be well to graduate them so as to cheapen the milk for infants under three months of age, in order to encourage mothers to bring their children early to the depôt. At Glasgow, where the lowest rates are charged, the price begins at one shilling per week; but perhaps even this might be made lower, with a corresponding increase in price for infants during the latter months of the first year.

(4) It is perhaps almost unnecessary to point out how much the work of visiting the mothers and tracing the progress of the infants would be facilitated by restricting the area of distribution. By means of a map kept at the depôt, the house of every infant supplied could be marked and seen at a glance, and by dividing up the area into a few sub-districts the work of the lady inspectors could be lightened, and at the same time could be carried out in a very thorough manner. This work has always appeared to me to be as important as the milk distribution itself, and it is one of the great advantages of the milk depôt that it renders this visiting and inspection possible in a way which nothing else can. The provision of milk becomes a basis for instruction of the mothers in general infant hygiene, and a means whereby they can be taught and advised in many matters outside the mere feeding of the children.[1]

[1] Since this article was written I have had an opportunity of reading Dr. G. F. McCleary's book, *Infantile Mortality and Infants' Milk Depôts.* On p. 102 he says, "The best way of applying this method (*i.e.* statistical comparison) is (1) to take a population small enough to be kept under observation; (2) to feed, from a given time, the whole of this population from the depôt; and (3) to compare the mortality before and after the introduction of the depôt milk, care being taken to extend the observations over a sufficiently long period, and to allow for meteorological variations," &c.

